# Carabidae diversity along an altitudinal gradient in a Peruvian cloud forest (Coleoptera)

**DOI:** 10.3897/zookeys.147.2047

**Published:** 2011-11-16

**Authors:** Sarah A. Maveety, Robert A. Browne, Terry L. Erwin

**Affiliations:** 1 Wake Forest University, Winston-Salem, NC, U.S.A.; 2 Department of Entomology, National Museum of Natural History, Smithsonian Institution, Washington, D.C., U.S.A.

**Keywords:** Ground beetles, tropical montane forests, Neotropics, pitfall traps, Andes

## Abstract

Carabid beetles were sampled at five sites, ranging from 1500 m to 3400 m, along a 15 km transect in the cloud forest of Manu National Park, Perú. Seasonal collections during a one year period yielded 77 morphospecies, of which 60% are projected to be undescribed species. There was a significant negative correlation between species richness and altitude, with the number of carabid species declining at the rate of one species for each 100 m increase in altitude. The majority of species (70.1 %) were restricted to only one altitudinal site and no species was found at more than three of the five altitudinal sites. Only one genus, *Pelmatellus* (Tribe Harpalini), was found at all five sites. Active (hand) collections yielded approximately twice as many species per individuals collected than passive (pitfall trap) collections. This study is the first systematic sampling ofcarabid beetles of a high altitude gradient in the cloud forests of southeastern Perú and supports the need to conserve the zone of extremely high biodiversity present on the eastern slopes of the Peruvian Andes.

## Introduction

Tropical forests may support up to 80 percent of the world’s biodiversity (Erwin 1996). In tropical montane cloud forests, moisture from the lowland forest rises and cools, enshrouding the area in heavy mist for at least part of each day (Lawton et al. 2001). With increasing altitude, temperature decreases, while condensation and precipitation increase, resulting in high humidity and relatively cool temperatures in montane cloud forests (Kricher 1997). Most high altitude tropical forests of the new world, including those found in the Andes Mountains, are dominated by cloud forests which support high levels of endemism and insularity (Schonberg et al. 2004). In the Andes Mountains of Perú, cloud forests generally exist at approximately 2000 to 3500 m (Kricher 1997) but can be locally variable, occurring as low as 1500 m in certain areas such as the K’osnipata Valley in southeastern Perú, the location of this study.

As with lowland rain forests, montane cloud forests are subject to numerous threats, including logging and land conversion to agriculture and pasturage (Kricher 1997; Schonberg et al. 2004); the destruction of nearby lowland tropical forest can also have an indirect and negative impact on montane cloud forests (Lawton et al. 2001). As climate change becomes more than just a prediction, cloud forests are expected to retreat with appropriate habitat shifting to higher, often anthropogenically disturbed altitudes, potentially affecting every organism within these highly fragile ecosystems (Nadkarni and Solano 2002; Pounds et al. 1999). Because of these expected transformations, research on cloud forest habitats can offer valuable insight on the effects of climate change.

Biological organisms are useful as indicators of local habitat change and as estimators forest fluctuations due to fragmentation or climate change (e.g., Jennings and Tallamy 2006; Chen et al. 2009), thus helpful for conservation biologists and policy makers. Understanding changes in biodiversity, due to direct causes such as land conversion, and indirect causes such as climate change, requires a baseline inventory. In the cloud forests of southeastern Perú, only limited data are available for biodiversity, and are further restricted to only a few taxa, such as birds (see Terborgh 1977), flowering plants (Gentry 1988a), and a few insect groups, e.g., wasps (Castillo-Cavero 2009) and ants (Azorsa-Salazar 2009). However, for one of the most diverse taxa, Coleoptera, baseline data are virtually non-existent for this critical habitat. Previously conducted tropical inventories have focused on lowland areas while data on montane areas is often lacking (Brehm et al. 2003; Schonberg et al. 2004). Species richness of many taxa is generally higher in the lower altitudes of a tropical altitudinal cline; e.g., trees (Kricher 1997), birds (Terborgh 1977), and insects (McCoy 1990; Escobar et al. 2005; Hanski and Niemela 1990). However, the number of endemic bird species in South America is approximately twice as high for high altitude cloud forests than adjacent lowland rain forests (Kricher 1997). Relatively little work has been done on the use of insects as biodiversity indicators in these tropical montane forests. More specifically, although a preliminary study of Carabidae community composition in the lowland Amazon Basin of Perú was reported by Erwin (1990), no studies have been conducted on the biodiversity of Carabidae on the highly diverse and imperiled tropical Andean slopes. Carabid beetles may be especially good biodiversity indicators because they not only can successfully signal environmental and ecological change (Niemelä et al. 2000, Desender et al. 1999) but they also vary widely in morphology, taxonomy, behavior and ecology (Erwin 1996). Carabidae are also easily collected and their identification for analysis is relatively uncomplicated (Erwin 1996). Importantly, carabid beetle collections appear to be representative of the composition of arthropod fauna in general (Butterfield et al. 1995).

In many areas of the tropics, where biodiversity is especially high but poorly inventoried, ecological researchers often encounter what has been termed the “taxonomic impediment” (New 1984, Cardosa et al. 2011), which is characterized by a difficulty in identification of species due to a lack of scientific names (Samways 1994). This “taxonomic impediment” can be overcome by continuing baseline studies in an attempt to catalog all diversity for future studies and synthesizing the objectives of taxonomy (e.g., inventory) and of ecological studies (e.g., uncovering patterns). However, as an intermediate step, the usual approach for ecological analysis is to classify new species as morphospecies. Given the lack of taxonomic keys for tropical, and especially Neotropical montane carabids, the morphospecies approach was utilized for the majority of the taxonomic work for this study. Since the effects of global climate change are expected to be the most severe for tropical insect communities, compared to their temperate counterparts, (Deutsch et al. 2008), inventory studies are especially needed in these areas.

In the present study, carabid beetles were collected from sites located on an altitudinal cline in the Andes Mountains in order to estimate absolute species diversity and extrapolated species richness. Incidence of rare and altitudinally restricted species is evaluated. Both active and passive collection methods were employed and the efficacy of these methods is compared.

## Methods

### Field study

**Figure 1.**
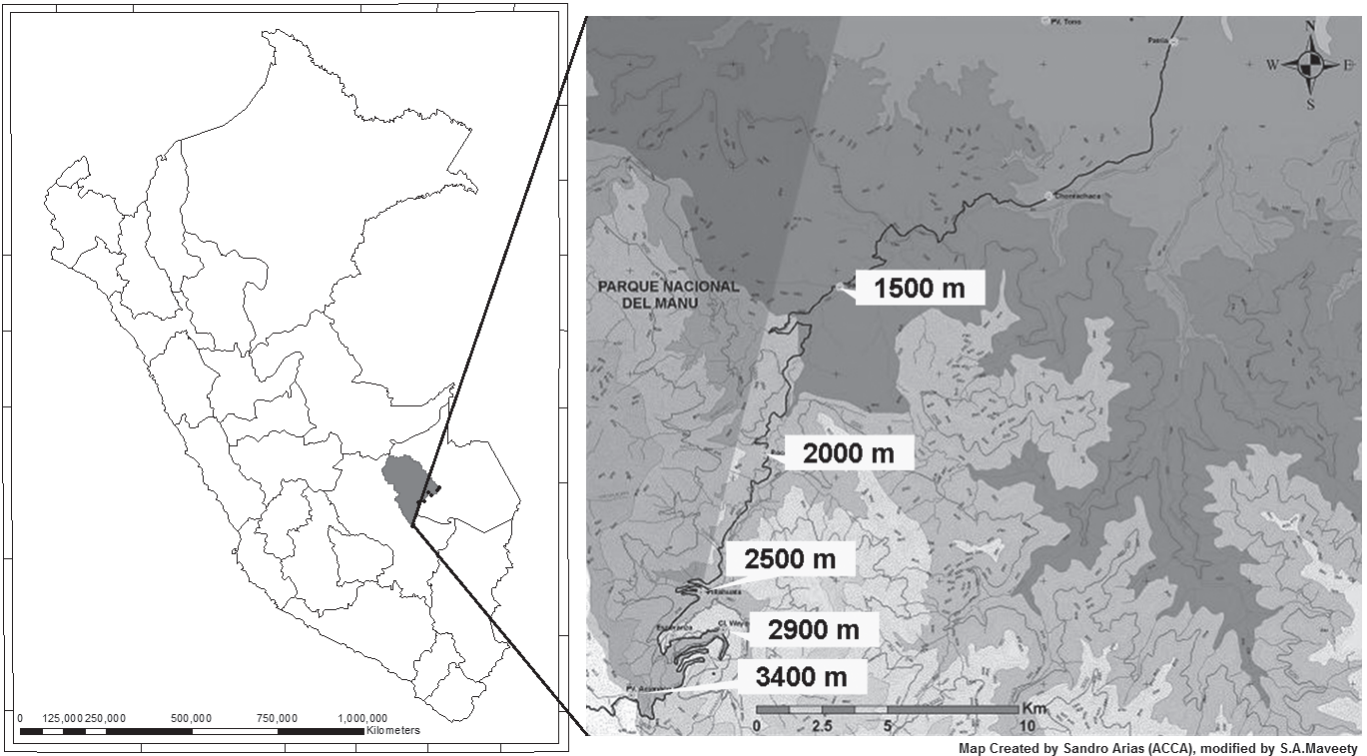
Map of collection sites.

Carabid beetles were collected at sites adjacent to the Cusco-Pilcopata highway in the Cultural Zone of Manu National Park, Department of Cusco, in southeastern Perú. Five sampling sites were established at approximately 500 m altitudinal intervals: Acjanaco Vigilante Point, (3400 m); Wayqecha Biological Station, (2900 m); Pillahuata Research , (2500 m); Rocotal, (2000 m); and San Pedro Biological Station, (1500 m). The transect decreases 1900 total meters over 15 km in space. Table 1 presents the GPS and altitude locality data for all sites. Geographical locations of the sites are depicted in Fig. 1.

**Table 1.** Locality data for the five altitudinal sites.

	**Altitude (m)**	**Altitude Band**	**Latitude (S)**	**Longitude (W)**	**No. Individuals**	**No. Genera**	**No. Species**
**AJANACO**	3470	3400	13°11.79'S, 71°37.22'W		162	3	7
**WAYQECHA**	–		–	–			
Trap A	2791	2900	13°10.99'S, 71°35.07'W		806	6	21
Trap B	2947		13°11.55'S, 71°35.28'W				
**PILLAHUATA**	–		–	–			
Trap A	2436	2500	13°09.69'S, 71°35.49'W		381	7	21
Trap B	2425		13°09.69'S, 71°35.47'W				
**ROCOTAL**	–		–	–			
Trap A	2084	2000	13°06.80'S, 71°34.25'W		227	13	28
Trap B	2082		13°06.68'S, 71°35.00'W				
**SAN PEDRO**	–		–	–			
Trap A	1432	1500	13°03.37'S, 71°32.83'W		348	12	28
Trap B	1427		13°03.37'S, 71°32.81'W				

At each site, carabid beetles were collected passively (via pitfall traps) and actively (via hand searches). Pitfall traps consisted of 1 liter plastic cups, the mouth inserted flush with ground level, with a plastic roof anchored by nails over the opening to prevent rain water from entering. Each trap was filled with salt water as a preservative and a small amount of soap to minimize surface tension. In order to account for possible microsite variation, two clusters of traps were placed at least 10 meters apart, with six traps per cluster for a total of 12 traps per site. Collections were conducted monthly from September 2007 to July 2008 by SAM and Peruvian assistants. Active collections consisted of sifting through leaf litter along the forest floor and examining above ground vegetation to approximately 1.5 m in height. Hand collections were done at night due to the generally nocturnal behavior of carabid beetles and included an additional collection during December 2008. All beetles were preserved in 95% ethanol. In order to provide an estimate of search effort, the number of collectors and the time spent searching was recorded for each site (see Table 1 of Online Material).

Since there were no previous collections in this area, a specific key to high altitude Neotropical carabid beetles and a list of species to this area were not available. Specimens where therefore identified to genus using a Neotropical Carabidae key (Reichardt 1977), a carabid beetle key to Pakitza, Manu N.P., Perú (Erwin 1990), and a key to tribes and genera of the Carabidae of Costa Rica (Erwin et al. 2004), and were subsequently identified to morphospecies level, with classification based on external morphology (not including genitalia). Identifications were made to morphospecies level by SAM with an original estimated 3% error rate, which was subsequently lowered via identifications at the Smithsonian Institution National Museum of Natural History by TLE.

## Data analysis

### Species Accumulation Curves

Species accumulation curves plot the number of species against the number of individuals in a sample and thus adjust species number for total sampling effort. As more individuals are collected only the rarest species are thought to be excluded from the collection; therefore, when all species are collected, the plotted line reaches a horizontal asymptote. Smoothed species accumulation curves were constructed using EstimateS 7.52 (Colwell 2005). Diversity estimators extrapolated by EstimateS 7.5.2 are as follows (for detailed descriptions of the richness estimators see Colwell (2005)): Mao Tau estimates the number of species expected in the total sample accumulated; A.C.E. (Abundance-based Coverage Estimator) is an estimator of species richness; Chao 1 is an additional richness estimator; Jack, a first-order Jackknife richness estimator; and Bootstrap, a bootstrap richness estimator. The following rarity estimates were also utilized from Colwell (2005): Singletons, the number of species with only one individual in total sample accumulated; Doubletons, number of species with only two individuals in total sample accumulated.

### Rarity

The number of rare species was estimated using both a taxonomic and an ecological index. The taxonomic index accounts for rare species as singletons (when a species is represented by one specimen) and doubletons, etc., up to n = 5. Such an index is more often used in studies of diversity from a taxonomic perspective (e.g., Coddington et al. 1991). Conversely the ecological index accounts for species rarity as a percentage of the total sample (e.g., Arscott et al. 2006). Rare species in this study are defined as ≤ 1% of the total number of individual collected (i.e., n = 19, based on a total of n = 1924 individuals), very rare species are represented by < 0.1% of the entire population (i.e., n ≤ 2). Singletons are defined as n = 1 and doubletons as n = 2.

### Ordination

Nonmetric Multidimensional Scaling (NMDS) in R 2.8.1 <www.r-project.org> was used for ordination analysis. Ordination analysis scores multivariate data in Euclidean space along two principle axes. NMDS uses a community similarity index to score data and places similar data points close in the ordination space. For all NMDS analyses Bray-Curtis similarity indices were used (Gotelli and Ellison 2004).

## Results

A total of 1,924 carabid beetle specimens were collected, represented by 13 tribes, 22 genera, and 77 morphospecies (Table 2). The most speciose tribes are Harpalini (represented by 27 morphospecies), Platynini (13 morphospecies) and Bembidiini (13 morphospecies). These three tribes also contain the largest number of collected individuals within a tribe: Harpalini (n = 1054), Platynini (n = 528), and Bembidiini (n = 118). Among the five sites the greatest number of individuals collected occurred at 2900 m (n = 802), with the least number of individuals at 3400 m (n = 162).

**Table 2.** List of tribes, genera and morphospecies of Carabidae collected.

**Tribe**	**Morphospecies**	**No.**	**Tribe**	**Morphospecies**	**No.**
Bembidiini	*Bembidion* A	5		*Pelmatellus* H	281
	*Bembidion* B	1		*Pelmatellus* I	8
	*Bembidion* C	12		*Pelmatellus* J	113
	*Bembidion* D	3		*Pelmatellus* K	15
	*Bembidion* E	25		*Pelmatellus* L	2
	*Bembidion* F	10		*Trichopselaphus* A	9
	*Bembidion* G	1		*Trichopselaphus* B	1
	*Bembidion* H	8	Lachnophorini	*Anchonoderus* A	12
	*Bembidion* I	10		*Psuedophoriticus* A	18
	*Bembidion* J	5		*Psuedophoriticus* B	2
	*Bembidion* K	6	Lebiini	*Calleida* A	3
	*Bembidion* L	25		*Calleida* B	1
	*Bembidion* M	7		*Lebia* A	1
Cicindelinae	*Psuedoxycheila lateguttata peruviana*	5<br/>	Oodini	*Lebia* B	1
				*Dercylus* A	1
Galeritiini	*Galerita* A	29	Ozaeniini	*Pachyteles* A	3
	*Galerita* B	3		*Pachyteles* B	2
	*Galerita* C	1		*Pachyteles* C	1
	*Galerita* D	2	Perigonini	*Diploharpus* A	1
Harpalini	*Goniocellus* A	1	Platynini	*Dyscolus* A	365
	*Notiobia* A	2		*Dyscolus* B	29
	*Notiobia* B	3		*Dyscolus* C	22
	*Notiobia* C	18		*Dyscolus* D	6
	*Notiobia* D	3		*Dyscolus* E	1
	*Notiobia* E	7		*Dyscolus* G	4
	*Notiobia* F	15		*Dyscolus* H	1
	*Notiobia* G	12		*Dyscolus* I	28
	*Notiobia* H	2		*Dyscolus* J	3
	*Notiobia* I	263		*Dyscolus* L	1
	*Notiobia* J	1		*Dyscolus* K	1
	*Notiobia* K	2		*Dyscolus* M	1
	*Notiobia* L	3		*Glyptolenus* A	66
	*Pelmatellus* A	1	Pterosticini	*Loxandrus* A	1
	*Pelmatellus* B	9		*Loxandrus* B	2
	*Pelmatellus* C	276		*Psuedobarys* A	1
	*Pelmatellus* D	3		*Trichonilla* A	1
	*Pelmatellus* E	1	Scaritini	*Ardistomus* A	3
	*Pelmatellus* F	2	Trechini	*Trechischibus* A	124
	*Pelmatellus* G	1		*Paratrechus sensulatt*	6

**Figure 2.**
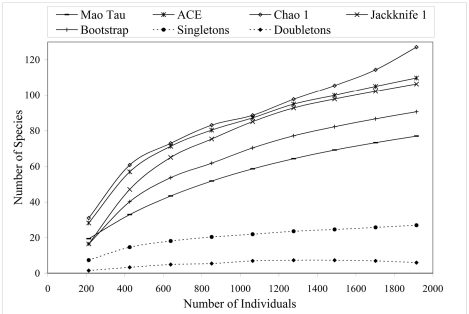
Species accumulation curves for 5 diversity indices when all altitudes and collection types (active or passive) are combined (n = 1924).

Figure 2 shows the species accumulation curve when all collection sites are combined (n = 1924) using the Mao Tau estimator (Colwell 2005). The accumulation curve approaches but does not fully reach asymptote. Extrapolation of the curve suggests that an asymptote could occur at approximately 3000 collected individuals, with a total of 90 species. Other methods for estimating diversity indices, namely A.C.E., Chao 1, Jack 1, and Bootstrap support the Mau Tau species estimate (Fig. 2), although at higher values for species number. All diversity indices suggest that species number did not reach asymptote at 2000 individuals collected. Fig. 2 also shows the species accumulation curve (when all sites are combined) for rarity categories more commonly utilized by taxonomists. Although the number of doubletons appears to asymptote at 1000 individuals the slope for singletons is positive up to the total number of individuals collected (1924).

**Figure 3.**
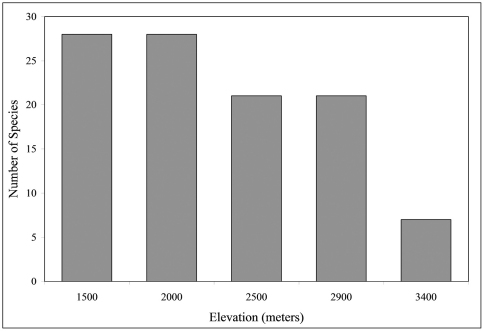
Number of species at five altitudes when all altitudes and collection types (active or passive) are combined (n = 1924).

As depicted in Fig. 3, the total number of species collected varied significantly with altitude (χ^2^ = 14.46, P < 0.01), with the highest species richness (S = 28) found at both 2000 m and 1500 m. There is a significant negative correlation between S and altitude (r = 0.91, P < 0.05). Regression analysis (y = –0.010x + 46.7, F_reg_ = 13.7, p < 0.05) indicates that the number of carabid species declines at the rate of 1.3% of the species diversity for each 100 m increase in altitude. Species numbers differed significantly (via χ^2 ^tests based on magnitude array) among altitudes as follows: 1500 m = 2000 m ≠ 2500 m = 2900 m ≠ 3400 m. Number of genera and tribes are also negatively correlated with altitude (r = 0.94, P <0.05; r = 0.91, P <0.05, respectively).

There was a high level of site specificity for species, genera and orders (Table 3). The majority of species (70.1 %; 54/77) were collected at only one altitudinal site. Only 6.5% (5/77) of the species were found at three altitudinal sites and no species was found at more than three sites. At higher levels of taxonomic organization, similar trends, though less pronounced, occurred, with 45.5 % (10/22) of genera and 38% (5/13) of tribes limited to one altitude site. One Tribe (Harpalini) and only one genus within (*Pelmatellus*) were found at all five sites.

**Table 3.** Site specificity by taxa (see text for additional descriptions).

**Number of Sites occupied**	**1**	**2**	**3**	**4**	**5**
Species	54	18	5	0	0
Genera	10	7	1	2	1
Tribes	5	3	2	2	1

**Figure 4.**
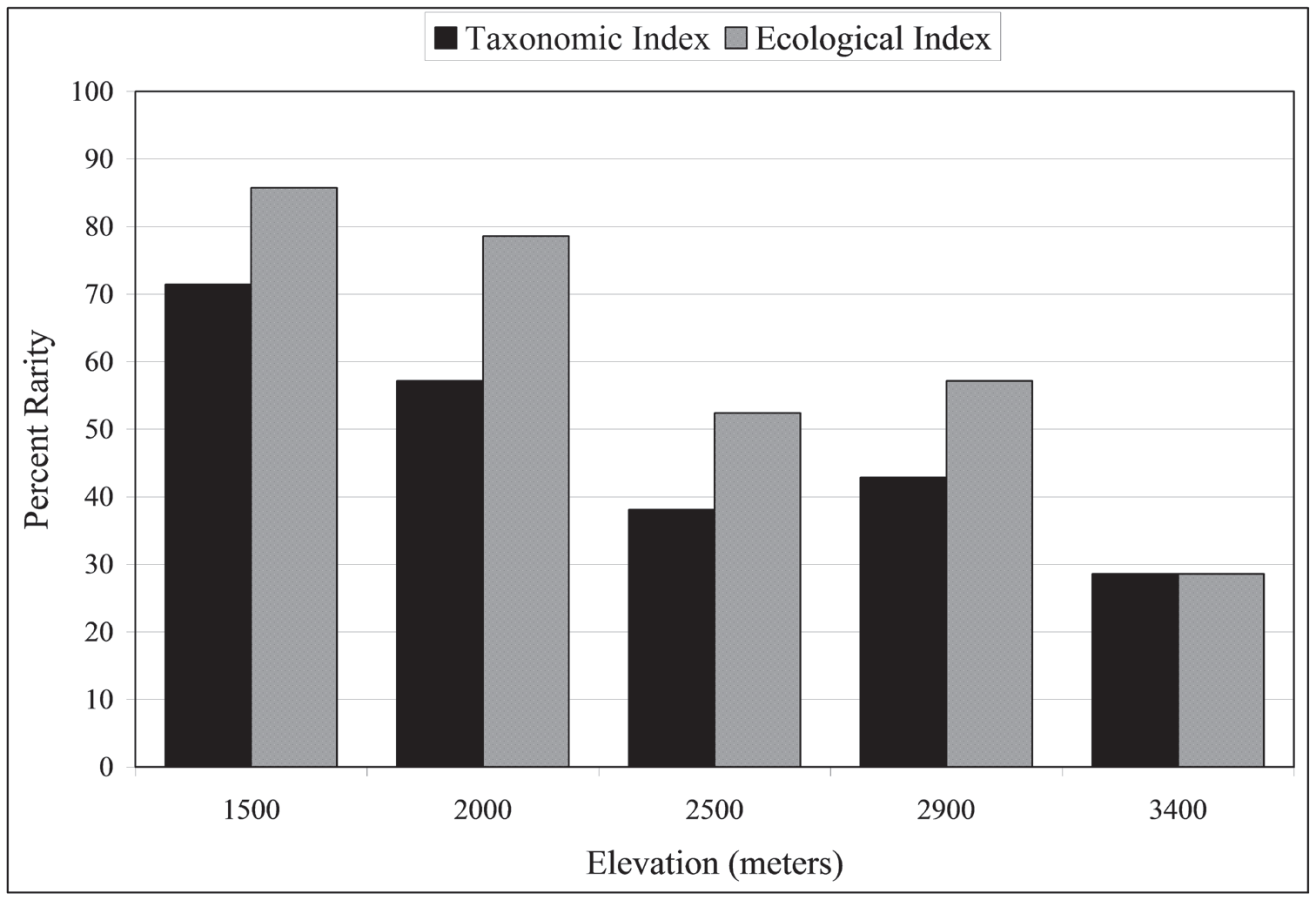
Percentage of rare species at five altitudinal sites. Black bars represent the taxonomic rarity index and gray bars represent the ecological rarity index (see text for definitions of rarity).

Rarity was estimated from both taxonomic and ecological perspectives (Fig. 4; see Materials and methods for how rarity is estimated). Rare species constitute 29% of the carabid beetle community at the highest altitude, 3400 m, and > 50% at both the two lowest altitudes. At 1500 m, 71% species are rare using the taxonomic index and 85.7% are rare by the ecological index. There are significant negative correlations with altitude for both rarity indices (r_tax_ = 0.951, r_eco_ = 0.953, P < 0.05).

**Figure 5.**
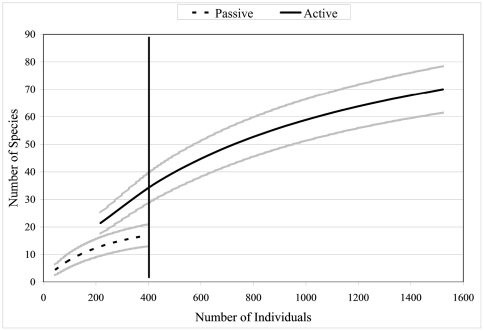
Species accumulation curves when all altitudes and collection types (active or passive) are combined (n = 1924). Brackets represent 95% confidence intervals.

**Figure 6.**
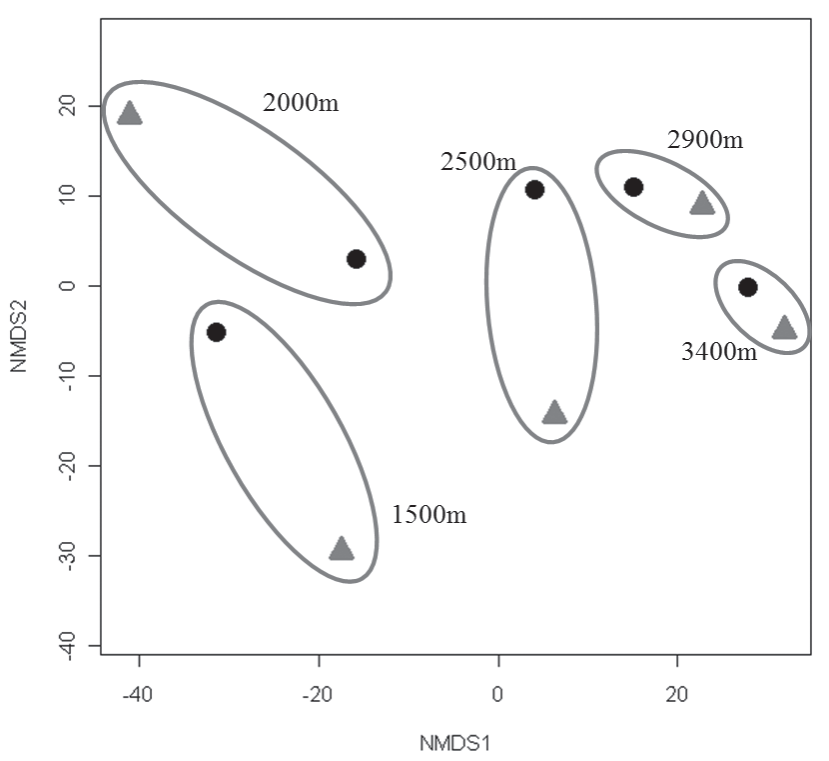
Non-Metric Multidimensional Scaling (NMDS) using the Bray Curtis Similarity Index for collections made actively (diamond) and passively (circle). Values for each altitude are grouped by ovals.

Since collections included both active (hand) and passive (pitfall trap) methods, comparisons can be made between the two techniques. Active collections yielded a greater number of species per individuals collected than passive collections (Fig. 5). Since more than three times the number of individuals was collected actively than obtained via passive collections, the number of species obtained would be expected to be higher for the former. However, the slope of the hand collection curve is also steeper than that generated from passive collections, with the non-overlapping 95% confidence intervals indicating that the number of species actively collected is significantly higher than for comparable size samples collected by passive techniques. For 400 individuals sampled (i.e., n= 400), S = 17 for passive collections while S = 37 for hand collections, suggesting that hand collecting detects more than twice as many species for the same number of individuals. When Nonmetric Multidimensional Scaling (NMDS) analysis is utilized (Fig. 6) the active and passive collection technique data points are not close together in space, suggesting that there is a slight compositional difference between carabid taxa collected actively or passively; however, the clustering of data points by altitudinal zone suggest a stronger altitudinal affect.

## Discussion

This study was the first inventory ofcarabid beetles for a high altitude gradient in the cloud forests of southeastern Perú. For approximately one year of collections, 77 morphospecies were collected. When more formal taxonomic analysis and a description of specimens are complete, approximately 60% of the species are expected to be previously undescribed (TLE). Further sampling will likely result in even more unknown species. Biodiversity inventories of altitudinal gradients are particularly relevant since they can serve as studies of climate change. For example, Chen et al. (2009) repeated a taxonomic inventory of Geometridae (Lepidoptera) in montane habitat in Borneo that had also been surveyed 40 years earlier. They found that average ranges of geometrid moths (102 species) had shifted upward by 67 m in altitude over the 40 year interval. Andean dung beetle altitudinal ranges have recently increased 72 m in the past 10 years (Larsen 2010). These increases could be due to various factors, but were most likely the result of a shift in the altitudinal zone due to a changing climate.

Species accumulation curves allow for direct comparison of S at the alpha level, thus avoiding some of the pitfalls with other diversity indicators, such as Shannon and Simpson indices (Veech and Crist 2010). The curves in the present analysis followed expected trends for tropical insects; when all samples are combined there is no evidence of asymptotic shape in the curve(s) indicating that the carabid beetle fauna has not been fully sampled with regard to the addition of new species. A continuously increasing species accumulation curve is expected for tropical insects (Escobar et al. 2005) because of the highly speciose nature of insects and the highly diverse nature of tropical regions.

As altitude increased, species diversity of carabid beetles decreased significantly. Tree diversity along the same altitudinal transect that was used in this study steadily increases from the Amazonian lowlands (approximately 500 m) to 1500 m, then decreases from 1500 m to 3400 m (Gentry 1988a; M. Silman pers comm.). Bird diversity parallels this change in vegetation diversity for altitudinal gradients in Perú (Terborgh 1977). Carabid beetle diversity may parallel the change in vegetation diversity on an altitudinal gradient as well, due to direct factors such as herbivorous carabid beetles or indirect factors such as carnivorous species. Species number also decreases with increasing altitude for dung beetles in tropical Borneo (Hanski and Niemelä 1990) and flying insects in Panama (Wolda 1987). An altitudinal study on temperate carabid fauna in Japan also reported a gradual decrease in species number along a gradient increasing in altitude (Hosoda 1999).

In addition to a significant change in species number with altitude, there is high degree of altitudinal site specificity, suggesting preferences for specific altitudes for the majority of carabid beetle species. Most species are confined to a single altitudinal site. The physical extremes and abrupt changes in abiotic conditions, such as a decrease in temperature and partial pressure of respiratory gases as well as an increase in precipitation (Hodkinson 2005), may restrict carabid beetle communities to distinct altitudinal zones. These changes result in a decrease in resource diversity, reduced habitat area, increase in unfavorable environment, and decrease in primary productivity (McCoy 1990). Biotic changes may also play a role. For example, the highest site, Acjanaco, at 3400 m, is near tree line, and the trees that are present are significantly smaller than those in lower altitudes. Carabid beetles at 3400 m may be adapted to these conditions and thus may be less adapted to forests with larger trees.Because of the absence and reduced size of trees, as well as abiotic factors, a smaller proportion of winged species would be expected at the highest altitudes (Darlington 1943; Poulsen 1996). A related study (Maveety and Browne in prep) indicates that the number of fully winged species is negatively correlated with altitude. Other studies in the Andes region have found that geographically restricted scarab beetles were negatively correlated with altitude (Escobar et al. 2005).

The dominant tribe collected in the study area was Harpalini; this group is composed of species that are mostly fully winged, and many Neotropical species are seed eaters (Arndt et al. 1996). Harpalini is one of the more speciose tribes of Carabidae (Cieglar 2000)and is represented in this collection by 2 genera, *Pelmatellus* and *Notiobia*. In the Neotropics, *Pelmatellus* is a high altitude genus with a body length of < 11mm while *Notiobia*’s body length is generally > 10 mm and inhabits lower altitudes (Goulet 1974; and Arndt (1998). The data from this study supports this observation since *Notiobia* was only found at the 1500 m and 2000 m sites, while *Pelmatellus* occurred along the entire gradient. Although sampling did not extend to altitude < 1500 m, we would speculate that 1500 m would be the lower limit for *Pelmatellus* while *Notiobia* and other genera would comprise the lowland Harpaline fauna at < 1500 m.

This was one of the first studies in a tropical montane environment to use both active and passive collecting techniques, which several studies suggest is necessary for complete inventories (e.g., Longino et al. 2002, Coddington et al. 1991). Estimates of S obtained from species accumulation curves suggested that active collecting produced almost twice as many species as did passive collecting for the same number of individuals sampled. Many carabid beetle studies, more often from temperate climates, employ pitfall trapping as it is a relatively simple and passive means of collection (Günther and Assman 2004; Liu et al. 2007). However, there are potential drawbacks associated with this technique. Pitfall traps may not accurately represent the true density of carabid fauna because they inherently measure species activity to represent species abundance (Greenslade 1964; Gutiérrez and Menéndez 1997; Lenski 1982). Nevertheless, Günther and Assmann (2004) have found that at least for two carabid beetle species there is a possible strong relationship between relative densities measured by pitfall traps and absolute density. Liu et al. (2007) disagrees, supporting that pitfall traps do not give a complete inventory of total carabid fauna present. This was clearly the case for carabid beetles collected in the present study, where more species were obtained from active collection than by passive pitfall traps (for the same sample size). Passive collections may only sample a small portion of the carabid beetle fauna in the tropics where carabid beetles fill a variety of niches including predators, herbivores, folivores, detritivores, scavengers, frugivores, wood-eaters, and grazers (Erwin 1979), behaviors that may not limit carabid beetles ground dwelling habitats. Species that are not often caught by pitfall traps are relatively easy to collect by active hand collection, as was the case in this study. The “duff” composition of the soils in the Andean cloud forests where there is a high level of non-decomposed organic material and numerous interstitial spaces among the material, which may allow carabids to travel below the nominal “soil” surface, may limit the effectiveness of pitfall trapping in this region.
